# Causal associations between CD40/CD40L and aortic diseases: A mendelian randomization study

**DOI:** 10.3389/fgene.2022.998525

**Published:** 2022-11-09

**Authors:** Xiao Cui, Tianming Xuan, Siyuan Chen, Xiaogang Guo

**Affiliations:** ^1^ Department of Cardiology, The First Affiliated Hospital, Zhejiang University School of Medicine, Hangzhou, China; ^2^ Graduate School, Zhejiang University School of Medicine, Hangzhou, China

**Keywords:** CD40, CD40L, aortic diseases, aortic dissection, aortic aneurysm, mendelian randomization

## Abstract

**Background:** CD40 and CD40L have been reported as associated with aortic dissection (AD) and aortic aneurysm (AA), but the causality of the associations has not been established yet.

**Methods:** We conducted a two-sample Mendelian randomization (MR) study to assess the causal inference between CD40/CD40L and aortic diseases including AD and AA. The instrumental variables (IVs) for CD40 and CD40L were selected from a high-quality protein quantitative trait loci dataset released by a genomic study involving 30,931 individuals of European ancestry. The genome-wide association studies summary statistics for AD and AA were from the FinnGen Release 7, with 288638 controls for all outcomes of interests, 680 cases for AD and 6,092 cases for AA, also from European ancestry. For AA subtypes, there were 5,881 cases of thoracic AA (TAA) and 2,434 cases of abdominal AA (AAA) respectively. Inverse-variance weighted and Wald ratio were applied for calculating causal estimates. Horizontal pleiotropy and heterogeneity were assessed using MR-Egger regression analysis and Cochran Q test, respectively. Leave-one-out analyses were further performed.

**Results:** Three single-nucleotide polymorphisms (SNPs) for CD40 and one SNP for CD40L were selected as IVs. We found genetic proxied CD40 levels inversely associated with the risk of AD (odds ratio [OR]: 0.777, 95% confidence interval [CI]: 0.618–0.978, *p* = 0.031) and AA (OR: 0.905, 95% CI: 0.837–0.978, *p* = 0.012), consistent across TAA (both *p* < 0.050). There were trends of increased risks of AD and AA in the presence of CD40L while not reaching statistical significance. No significant horizontal pleiotropy or heterogeneity was observed.

**Conclusion:** Our MR study provides evidence supporting the causal association between CD40 and the reduced risks of both AD and AA.

## Introduction

Aortic dissection (AD) and aortic aneurysm (AA) are severe aortic diseases with life-threatening risks (Erbel et al., 2014; Bossone and Eagle 2021). AD, defined as disruption of the medial layer provoked by intramural bleeding, resulting in separation of the aortic wall layers and subsequent formation of a true lumen and a false lumen with or without communication, presents with very high mortality (Erbel et al., 2014). Aortic aneurysm, including thoracic aortic aneurysm (TAA) and abdominal aortic aneurysm (AAA), though might be asymptomatic for years, is also fatal when the cataclysmic dissection or rupture occurred (Erbel et al., 2014). Despite recent scientific advances in both AD and AA, the underlying mechanisms have not been fully understood. Moreover, there is still a lack of effective medications for preventing the development or progression of these devastating diseases (Shen et al., 2020a; 2020b).

CD40 and its ligand CD40L (CD154), members of the tumour necrosis factor receptor (TNFR) and tumour necrosis factor (TNF) ligands superfamily, play crucial roles in both humoral and cellular immune responses (Tang et al., 2021). CD40 and CD40L are widely expressed in various cell types, among which are constitutively expressed on B cells and dendritic cells, and expressed on T cells and macrophages after cellular activation. They are also expressed on many non-immune cells, such as endothelial cells, vascular smooth muscle cells and fibroblasts (Tang et al., 2021). Blocking the interaction between CD40/CD40L, using genetic or pharmacological (i.e., monoclonal antibody) means, has been implicated in various autoimmune diseases such as rheumatoid arthritis (Karnell et al., 2019). On the opposite side, the stimulation of CD40 through agonistic antibodies has been regarded as a promising approach for cancer treatment (Bullock 2022).

Inflammation is well-recognized as a risk factor for cardiovascular diseases. In line with this, previous evidence has indicated that CD40 and CD40L are closely related to cardiovascular diseases (Heeschen et al., 2003; Ueland et al., 2005; Hausding et al., 2013; Daub et al., 2020; Sultan et al., 2020). The role of CD40/CD40L in atherosclerosis, particularly in atherothrombosis, has been well-studied (Antoniades et al., 2009; Michel et al., 2017). Observational studies in patients with aortic dissection and aortic aneurysm have found elevated plasma levels of soluble CD40L (Touat et al., 2006; Vianello et al., 2016). Microarray-based gene expression analyses of human aortic tissue samples further revealed overexpression of both CD40 and CD40L in the thrombus-free and thrombus-covered AAA aortic media (Kusters et al., 2018). Experimental studies using the mice models further proved that CD40L participates in the pathological process of AD and AA (Han et al., 2018; Kusters et al., 2018). Genetic depletion of CD40L in a β-aminopropionitrile-induced mouse model failed to develop acute AD (Han et al., 2018). Also, deficiency of hematopoietic CD40L in mice was shown to protect against dissecting aneurysm formation and reduces the incidence of fatal rupture (Kusters et al., 2018). These observational and experimental findings suggest CD40/CD40L as a potential target for AD and AA. But it is still far from clinical translation.

Moreover, safety concerns further limit the conduction of clinical trials targeting CD40/CD40L in patients with AD and AA. The immunosuppression effects brought by the blockade of CD40/CD40L might make patients exposed to high risks of severe infection and cancers. Moreover, there were also reports of unexpected thromboembolic events in anti-CD40L pre-clinical and clinical trials, indicating the role of CD40L in thrombus stabilization might be a double-edged sword. An unusually high incidence of thromboembolic complications was observed in monkeys treated with a monoclonal antibody against CD40 ligand (Kawai et al., 2000). Large thrombi developed in CD40L-deficient mice were prone to rupture and embolization (André et al., 2002). A clinical trial of a humanized anti-CD40L antibody in patients with proliferative lupus glomerulonephritis had to be terminated prematurely because of thromboembolic events occurring in patients (Boumpas et al., 2003). Given such controversy between potential therapeutic values and non-negligible life-threatening risks, it is still too early for conducting large-scale randomized controlled clinical trials regarding the effects of CD40/CD40L on AD and AA. Alternatively, Mendelian randomization (MR) analysis, a novel epidemiological method based on large-scale human genetic summary statistics, could provide us with a feasible and safe way for that purpose. The rationale of MR is by using instrumental variables (IVs), mostly single-nucleotide polymorphisms (SNPs) identified from genome-wide association studies (GWASs), as proxies to explore the causal inferences between the exposures and the diseases of interest, and consequently less biased by confounding factors and reverse causation (Emdin et al., 2017). Hence, by leveraging the summary statistics of the most recent large-scale protein quantitative trait loci (pQTL) and GWAS datasets, we conducted a two-sample MR study for the causal inference between CD40/CD40L and aortic diseases including AD and AA, aiming to assess whether causality exists or not and whether the effect is to promote or prevent.

## Materials and Methods

### Study design

**FIGURE 1:**
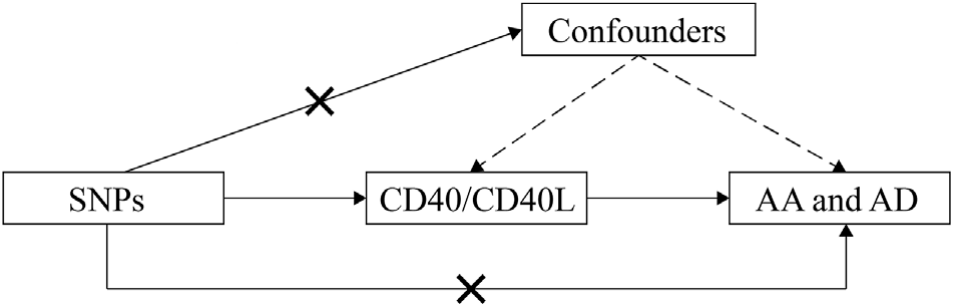
Schematic representation of this MR study. SNPs, single-nucleotide polymorphisms; AD, aortic dissection; AA, aortic aneurysm; MR, Mendelian randomization.

Using summary data from published studies and corresponding resources as described in detail as follows, we conducted this two-sample MR study to assess the causal inference betweenCD40/CD40L and aortic diseases including AD and AA (Figure 1).

### Selection of instrumental variables

The instrumental variables (IVs) for CD40 and CD40L were selected from a high-quality pQTL dataset released by a genomic and drug target evaluation study for 90 cardiovascular proteins involving 30,931 individuals of European ancestry (http://www.scallop-consortium.com/) (Folkersen et al., 2020). All selected IVs met the three stringent assumptions required for MR analysis: 1) the IVs are associated with the modifiable exposure of interest; 2) the IVs are independent of the confounding factors associated between exposure and outcome; 3) any association between the instruments and the outcome is mediated by the exposure (Burgess et al., 2017). To avoid potential pleiotropic effect on causality estimates, selected SNPs were manually scanned with the PhenoScanner (http://www.phenoscanner.medschl.cam. ac.uk), using a *p*-value of 1 × 10^–5^ and R^2^ of 0.8 as thresholds. To assess correlated alleles for a pair of variants in high LD, we used the LDpair module in LDlink (Machiela and Chanock 2015).

### Data sources of outcomes

The GWAS summary statistics for AD and AA are from the FinnGen Release 7, one of the very first personalized medicine projects aiming to identify genotype-phenotype correlations through collecting and analysing genome and health data from Finnish biobank participants (https://www.finngen.fi/en). The analytical process of GWAS data is described in detail in the following link (https://finngen.gitbook.io/documentation/v/r7/methods/phewas). The covariates used in the model include sex, age, 10 principal components and genotyping batch. The study population consists of 288638 controls for all outcomes of interests, 680 cases for AD and 6,092 cases for AA. For AA subtypes, there were 5,881 cases of TAA and 2,434 cases of AAA, respectively (https://finngen.gitbook.io/documentation/v/r7/). AD and AA as endpoints were defined using nationwide registries, harmonized over the International Classification of Diseases (ICD) revisions 8, 9 and 10, cancer-specific ICD-O-3, (NOMESCO) procedure codes, Finnish-specific Social Insurance Institute (KELA) drug reimbursement codes and ATC-codes (https://www.finngen.fi/en/researchers/clinical-endpoints).

### Statistical analysis

All estimates were two-sided at 0.05 as statistical significance. F-statistics from the first-stage regression of the exposure phenotype on IVs were calculated to evaluate weak instruments using the approximation method (Bowden et al., 2016). Two-sample MR analysis was conducted with the inverse-variance weighted (IVW) method based on the fixed-effects model as the number of IVs ≤3. For MR analysis with only one IV, the Wald ratio was presented. To test the overall unbalanced horizontal pleiotropy, the MR-Egger regression analysis was performed. The Cochran Q test was used for heterogeneity testing with a *p*-value < 0.05 considered as significantly heterogeneous. In addition, the leave-one-out analyses were applied to ensure the reliability of the results. All statistical data analyses were conducted with R software, version 4.2.1 (http://www.r-project.org), using the TwoSampleMR and MRPRESSO packages.

## Results

### Basic characteristics of instrumental variables

Based on the methods as described in the Materials and Methods section, we finally selected three SNPs as IVs for CD40 and one SNP for CD40L (Table 1). In the study, independent (LD R^2^ = 0) *cis*- and *trans*-pQTLs were selected as genetic proxies for the CD40 and CD40L. The two SNPs showing *cis*-regulatory effects on CD40 (rs4810485 and rs41282788) are in linkage equilibrium, according to the analysis result using the LDpair module in the LDlink. The approximate F-statistics from the first-stage regression of the exposure phenotype CD40/CD40L on SNPs ranged from 29.469 to 1736.111, ensuring sufficient strength for estimating causal associations.

**TABLE 1:** SNPs used as IVs for MR analyses.

Exposure	SNP	*cis*/*trans*	EA	OA	EAF	beta	Se	*p*-value	appF
CD40	rs4801216	*trans*	T	C	0.600	-0.090	0.013	1.80E-12	47.929
CD40	rs4810485	*cis*	T	G	0.260	-0.500	0.012	2.3E-392	1736.111
CD40	rs41282788	*cis*	C	G	0.022	0.540	0.042	2.60E-38	165.306
CD40-L	rs4602861	*trans*	A	G	0.750	0.076	0.014	1.40E-08	29.469

SNPs, single-nucleotide polymorphisms; IVs, instrumental variables; MR, mendelian randomization; EA, effect allele; OA, other alleles; EAF, effect allele frequency.

### Genetic proxied CD40/CD40L levels on the risk of aortic dissection

**FIGURE 2:**
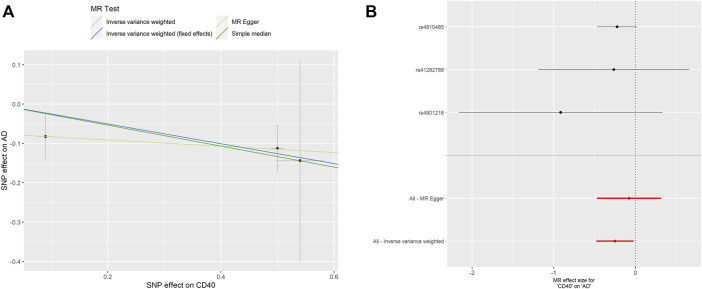
MR analyses of genetic proxied CD40 levels on the risk of AD. The scatterplot **(A)** and the forest plot **(B)** of the MR analysis of CD40 and AD. AD, aortic dissection; MR, Mendelian randomization.

As shown in Table 2, surprisingly, we found genetic proxied CD40 levels inversely associated with the risk of AD (odds ratio [OR]: 0.777, 95% confidence interval [CI]: 0.618–0.978, *p* = 0.031). No significant horizontal pleiotropy was observed according to the MR-Egger regression analysis (*p* = 0.482, Supplementary Table S1). No significant heterogeneity was present according to Cochran’s Q test (*p* = 0.569, Supplementary Table S2), either. The effective value of individual IVs of CD40 on AD was demonstrated by scatterplot and forest plot shown in Figure 2. However, analysis of genetic proxied CD40L levels on the risk of AD did not reach statistical significance (OR: 2.779, 95%CI: 0.508–15.205, *p* = 0.239, Table 2). The leave-on-out analysis of CD40 on AD was presented in Supplementary Figure S1.

**TABLE 2:** Two-sample MR analyses of associations between CD40/CD40L and aortic diseases.

Exposure	Outcome	Method	nSNP	OR	95% CI	*p*-Value
CD40	AD	IVW	3	0.777	0.618–0.978	0.031
CD40	AA	IVW	3	0.905	0.837–0.978	0.012
CD40	TAA	IVW	3	0.906	0.837–0.981	0.015
CD40	AAA	IVW	3	0.851	0.753–0.962	0.010
CD40-L	AD	Wald ratio	1	2.779	0.508–15.205	0.239
CD40-L	AA	Wald ratio	1	1.091	0.608–1.957	0.770
CD40-L	TAA	Wald ratio	1	1.116	0.616–2.020	0.718
CD40-L	AAA	Wald ratio	1	1.029	0.410–2.582	0.952

MR, mendelian randomization; SNPs, single nucleotide polymorphisms; AD, aortic dissection; AA, aortic aneurysm; TAA, thoracic aortic aneurysm; AAA, abdominal aortic aneurysm; IVW, inverse variance weighted (fixed effects); OR, odds ratio; CI, confidence interval.

### Genetic proxied CD40/CD40L levels on the risk of aortic aneurysm

**FIGURE 3:**
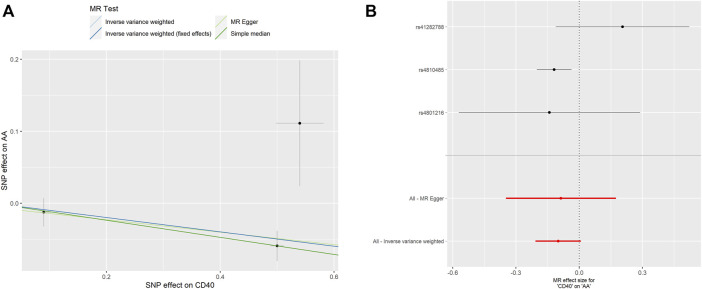
MR analyses of genetic proxied CD40 levels on the risk of AA. The scatterplot **(A)** and the forest plot **(B)** of the MR analysis of CD40 and AA. AA, aortic aneurysm; MR, Mendelian randomization.

As shown in Table 2, the genetic proxied CD40 level is inversely associated with the risk of AA (OR: 0.905, 95% CI: 0.837–0.978, *p* = 0.012). No significant horizontal pleiotropy (*p* = 0.923) or heterogeneity (*p* = 0.149) was observed (Supplementary Tables S1, S2). The effective values of CD40 and AA were visualized using the scatter plot and forest plots as shown in Figure 3. However, analysis of genetic proxied CD40L levels on the risk of AA did not reach statistical significance (OR: 1.091, 95%CI: 0.608–1.957, *p* = 0.770, Table 2), either. The leave-on-out analysis of CD40 on AA was presented in Supplementary Figure S2.

### Genetic proxied CD40/CD40L levels on the risk of aortic aneurysm subtypes

**FIGURE 4:**
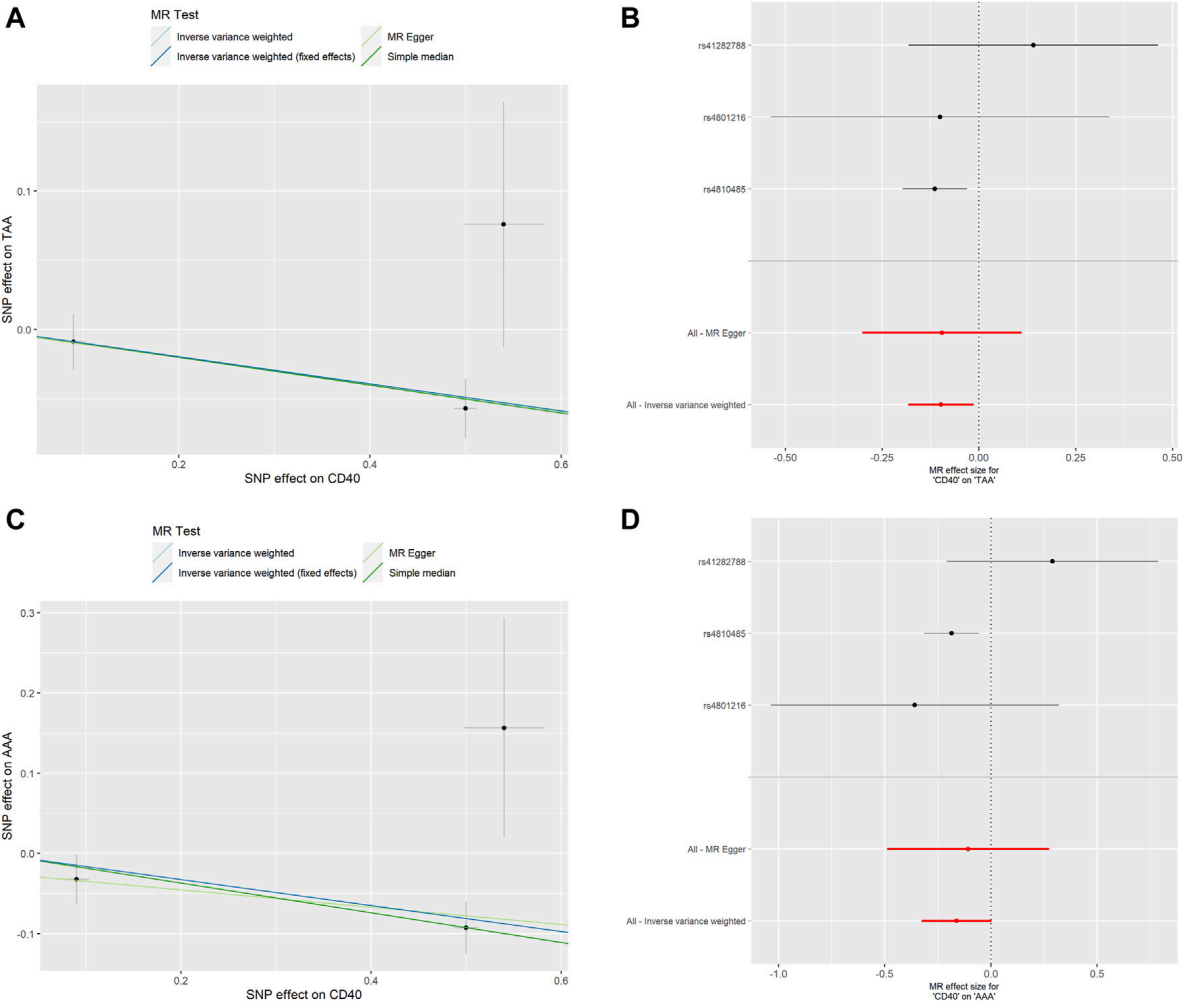
MR analyses of genetic proxied CD40 levels on the risk of AA subtypes. The scatterplot **(A)** and the forest plot **(B)** of the MR analysis of CD40 and TAA. The scatterplot **(C)** and the forest plot **(D)** of the MR analysis of CD40 and AAA. AA, aortic aneurysm; TAA, thoracic aortic aneurysm; AAA, abdominal aortic aneurysm; MR, Mendelian randomization.

To assess whether the relationships between CD40/CD40L and AA were consistent across the subtypes of AA, we further examine the potential effects of CD40/CD40L on TAA and AAA, respectively. As shown in Table 2, we also found that genetic proxied CD40 levels demonstrate inverse associations with the risk of TAA (OR: 0.906, 95% CI: 0.837–0.981, *p* = 0.015) and AAA (OR: 0.851, 95% CI: 0.753–0.962, *p* = 0.010). No significant horizontal pleiotropy (TAA, *p* = 0.982; AAA, *p* = 0.790) or heterogeneity was present (TAA, *p* = 0.324; AAA, *p* = 0.163), either (Supplementary Tables S1, S2). Figure 4 shows the scatter plot and forest plots of CD40 and the two AA subtypes. Still, the causality assessment between genetic proxied CD40L and the two AA subtypes did not reach statistical significance, either (TAA, OR: 1.116, 95%CI: 0.616–2.020, *p* = 0.718; AAA, OR: 1.029, 95%CI: 0.410–2.582, *p* = 0.952, Table 2). The leave-on-out analysis was presented in Supplementary Figures S3, S4.

## Discussion

Using a two-sample MR approach with independent IVs, we confirmed causal associations between CD40 and two important aortic diseases, AD and AA, in a surprisingly inverse direction, that CD40 was causally associated with reduced risks of AD and AA. The estimated effects of genetic proxied CD40 on AA were consistent across AA subtypes.

Previous studies have indicated CD40/CD40L is associated with AD and AA, while insufficient for ensuring a causal inference. In a pilot case-control study measuring levels of several circulating inflammatory molecules, researchers found that both type A and B acute AD patients had significantly higher CD40L levels within 24 h of symptom onset (Vianello et al., 2016). Soluble CD40L, along with other platelet activation markers, has also been found to increase in the plasma from patients with AAA, and in eluates from the luminal thrombus layer in the experimental AAA rat model (Touat et al., 2006). CD40 and CD40L gene upregulation in the aortic tissue samples from AAA patients were also observed (Kusters et al., 2018). However, these observational associations are unable to provide a causal inference due to the nature of the study design. Although some animal experiments have suggested the involvement of CD40L in AD and AA, human data for assessing their effects on AD and AA is still lacking. As previous studies suggest CD40L positively correlated with AA and AD, we also find the correlations between them in the same direction, that there were trends of increased risks of AD and AA in the presence of CD40L according to our MR analyses. But these correlations did not reach statistical significance. Meanwhile, as we could easily notice, most of these previous studies focused on CD40L rather than CD40. Even though CD40L is the ligand of CD40, their effects should not be mixed up. The role of CD40L on aortic diseases does not implicate that of CD40. Evidence regarding CD40 and aortic diseases is insufficient. In our present study, we revealed significantly reduced risks of AD and AA in the presence of higher CD40 levels, while a trend of increased risks of AD and AA in the presence of higher CD40L levels. These results suggested that CD40 and CD40L might exert a distinct pathogenic role in the mechanisms of AD and AA.

Since CD40/CD40L are essential components in both innate and acquired immunity, monoclonal antibodies targeting CD40/CD40L, either antagonistic or agonistic, have attracted widespread attention in the field of autoimmune diseases and cancers, respectively (Karnell et al., 2019; Bullock 2022). There were already randomized controlled trials supporting the therapeutic value of antagonistic anti-CD40 monoclonal antibodies in autoimmune diseases such as rheumatoid arthritis and graves hyperthyroidism (Espié et al., 2020; Kahaly et al., 2020). However, despite of beneficial effects of blocking CD40 in treating these diseases, safety issues should also be concerned. The interaction of soluble CD40L with β3 integrins could affect the stability of thrombi, which might complicate clinical outcomes of occlusion and embolization (André et al., 2002). As mentioned in the Introduction section, researchers unexpectedly observed an increased occurrence of life-threatening thromboembolic events when blocking CD40L, although there were also trials observing no thromboembolic events in subjects receiving monoclonal antibody against CD40L from another manufacturer (Kawai et al., 2000; Kalunian et al., 2002; Boumpas et al., 2003; Fadul et al., 2021). Considering these controversial results, along with the wide and complicated involvement of CD40/C40L in physiology and pathophysiology, beyond the already known risks of infection and cancer, we should also be alert to other potential risks when administering patients with medications targeting CD40/C40L. Our present MR study shows reduced risks of AD and AA in the presence of higher CD40 levels. Hence, when administering patients with CD40-blockade therapy for treating autoimmune diseases, whether it will be accompanied by higher risks of developing AD and AA? This is worth keeping a watchful eye on.

In the present MR study, independent *cis*- and *trans*-pQTLs were selected as genetic proxies for the CD40 and CD40L. For CD40, two *cis*-regulatory and one *trans*-regulatory SNPs were selected as IV. The *cis*-regulatory elements are adjacent to the coding gene transcriptional start site and most of the time reside in the promoter or enhancer regions. The promoter-enhancer loop settled by different transcriptional machinery is crucial to initiate transcription and determines the level of transcriptional activity (Moore et al., 2020). However, the function of *trans*-regulatory elements, sometimes, would be hard to explain. In this case, the rs4801216 that shown to play *trans*-regulatory effects on the CD40 is considered as the leading SNP of zinc finger protein 543 (ZNF543), which serves as a transcription factor. Although there is no data available about the relationship between ZNF543 and CD40 for aortic vasculature cells, there was data indicating ZNF543 binding in the promoter region of CD40 in 293T cells, suggesting that the ZNF543 might either promote or inhibit CD40 expression by directly binding to the promoter region of CD40 (Imbeault et al., 2017). A more definite way to confirm this is to isolate the cell type of interest and perform chromatin immunoprecipitation sequencing (ChIP-seq) or cleavage under targets and tagmentation (CUT&Tag). There are several limitations of this study. First, although we examine the effects of genetic proxied CD40/CD40L levels in AA subtypes, we, unfortunately, could not evaluate that in AD subtypes due to the lack of corresponding summary-level datasets. Second, the study population were of European ancestry, which limits the generalization of our findings to other populations. Third, the fact that the association between CD40L and higher risks of AD and AA have not achieved statistical significance cannot enable us to rule out potential causal effects, as the negative results in an MR analysis might be due to the insufficient power of IVs and relatively small sample sizes. Fourth, it should be noted that genetic changes may not translate into the functional form. Other methodologies are warranted before translating these findings to clinical usage. Furthermore, due to the sample size, we are not able to identify other SNPs that could either function in *cis*- or *trans*-format, but it is possible that some SNPs that are weakly associated with post-transcriptional or post-translational regulation could play a role in generating more functional CD40. Likewise, SNPs associated with protein degradation machinery could also play a role. Follow-up studies with larger sample sizes are needed to confirm our hypothesis.

Taken together, this MR study provides genetic-based evidence supporting the causal association between CD40 and the reduced risks of both AD and AA, suggesting the value of CD40 as a prediction marker for these two severe diseases. To some extent, it also proposes the therapeutic potentiality of CD40 agonistic therapy for AD and AA. However, these findings are still far from clinical interpretation and application. More studies are needed to further reveal the role of CD40 on AD and AA.

## Data Availability

Publicly available datasets were analyzed in this study. This data can be found here: SCALLOP CVD-I online resource (http://www.scallop-consortium.com/) and FinnGen release 7 (https://finngen.gitbook.io/documentation/v/r7/).
