# Supplementary material for: Causal associations between CD40/CD40L and aortic diseases: A mendelian randomization study
Source: Front Genet. 2022 Nov 9;13:998525. doi: 10.3389/fgene.2022.998525 (PMC9681816; doi:10.3389/fgene.2022.998525)
Supplement: Supplementary file 1 [file DataSheet1.PDF]

Supplementary Material

Supplementary Figures and Tables

Supplementary Figures

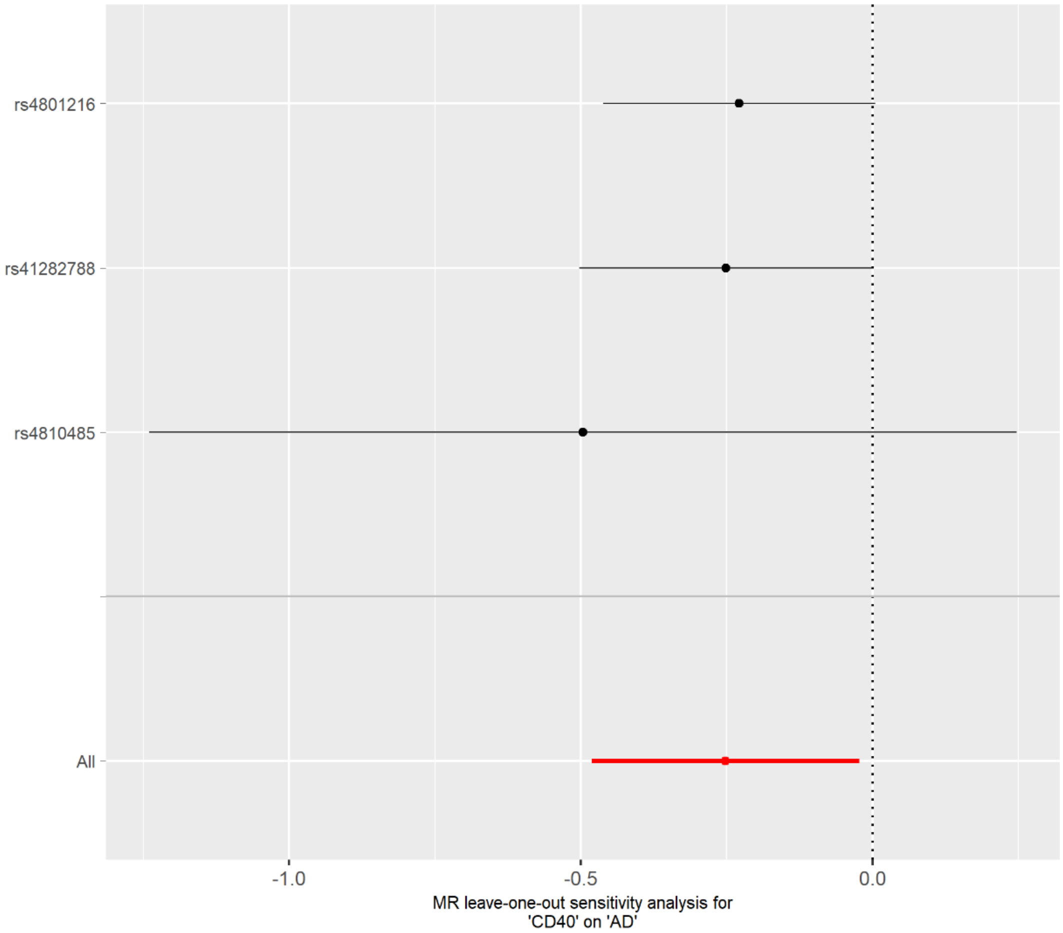

Supplemental Figure 1. Leave-one-out sensitivity analysis of genetic proxied CD40 levels on the risk of AD. AD, aortic dissection; MR, Mendelian randomization.

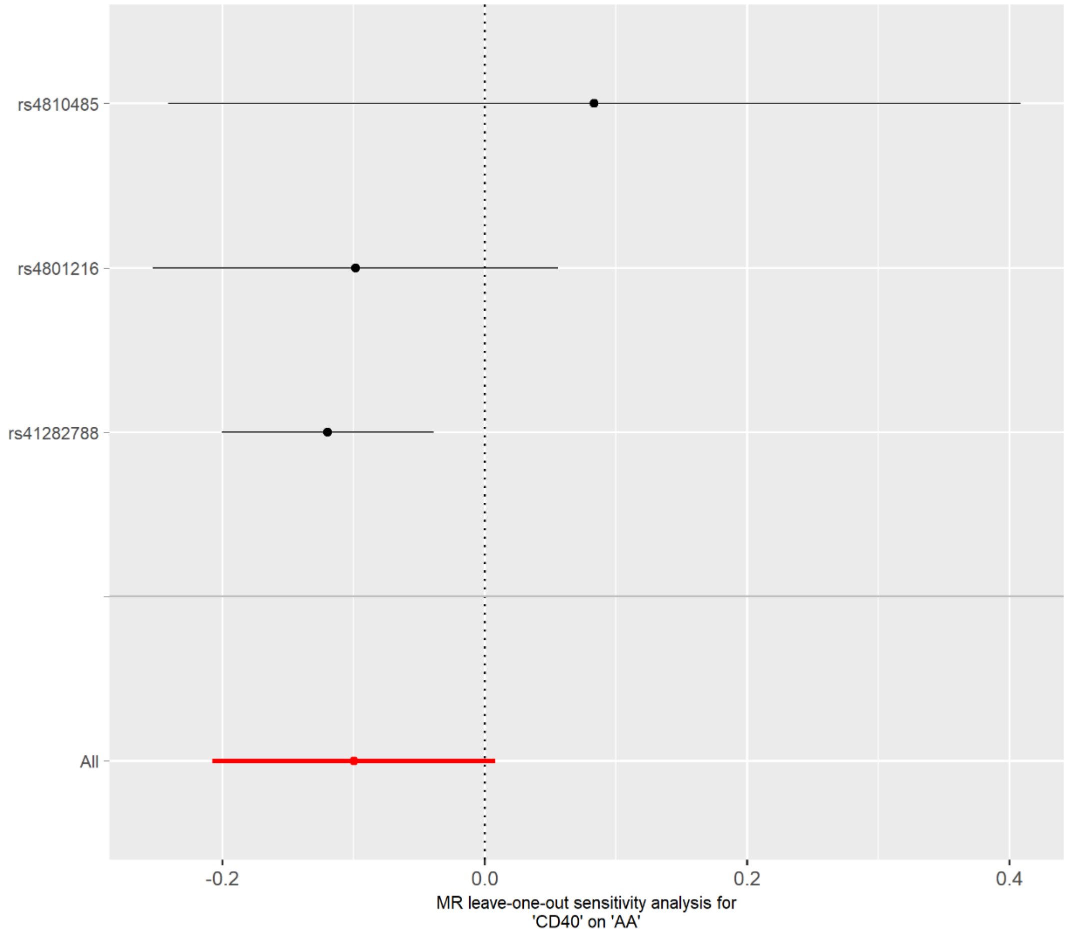

Supplemental Figure 2. Leave-one-out sensitivity analysis of genetic proxied CD40 levels on the risk of AA. AA, aortic aneurysm; MR, Mendelian randomization.

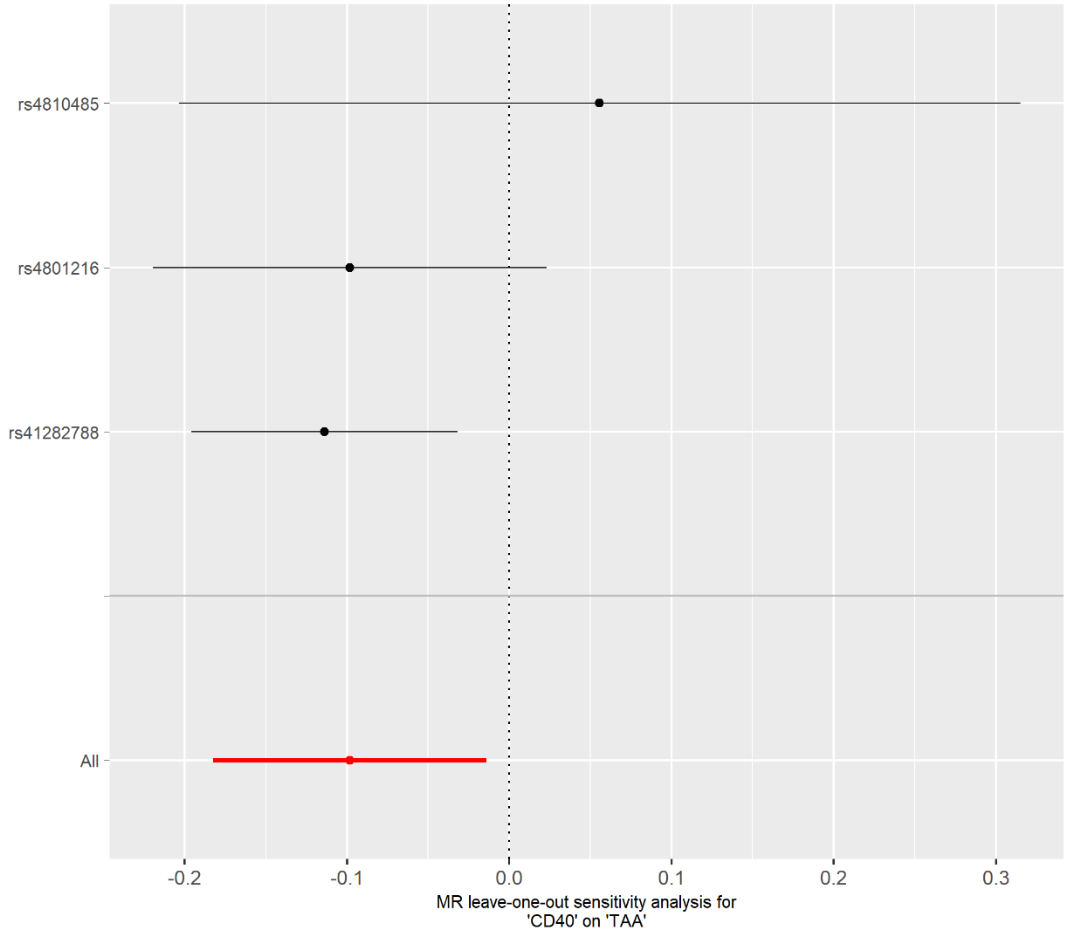

Supplemental Figure 3. Leave-one-out sensitivity analysis of genetic proxied CD40 levels on the risk of TAA. TAA, thoracic aortic aneurysm; MR, Mendelian randomization.

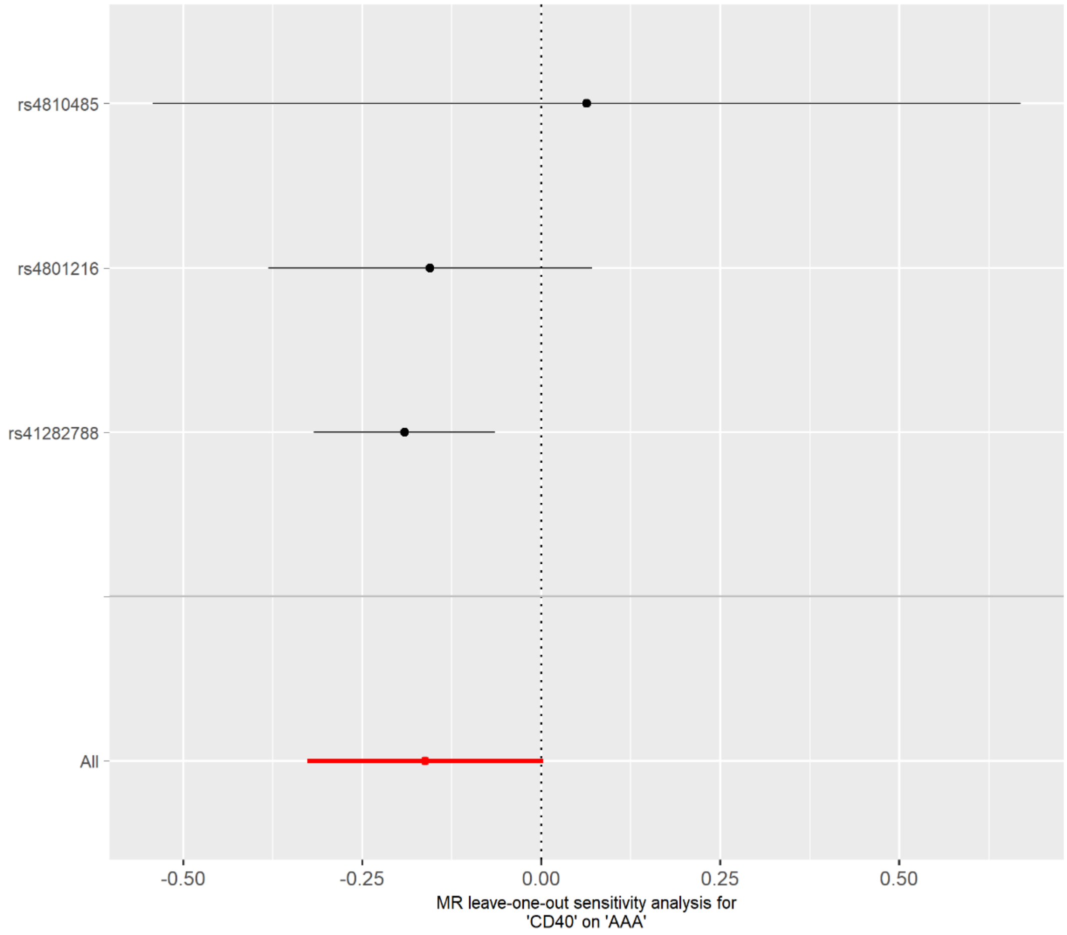

Supplemental Figure 4 Leave-one-out sensitivity analysis of genetic proxied CD40 levels on the risk of AAA. AAA, abdominal aortic aneurysm; MR, Mendelian randomization.

Supplementary Tables

Supplementary Table 1. Pleiotropy analyses based on MR-Egger regression of the two-sample MR analyses of CD40 and aortic diseases

Aortic diseases	Intercept	Se	P value
AD	-0.075	0.071	0.482
AA	-0.006	0.047	0.923
TAA	-0.001	0.037	0.982

AAA	-0.024	0.069	0.790

AA, aortic aneurysm; TAA, thoracic aortic aneurysm; AAA, abdominal aortic aneurysm.

Supplementary Table 2. Heterogeneity analyses based on Cochrane's Q test for the two-sample MR analyses of CD40 and aortic diseases

Aortic diseases	Cochrane's Q	Qdf	H statistic	I^2	P value
AD	1.129	2	0.751	-0.772	0.569
AA	3.814	2	1.381	0.476	0.149
TAA	2.253	2	1.061	0.112	0.324
AAA	3.622	2	1.346	0.448	0.163

AA, aortic aneurysm; TAA, thoracic aortic aneurysm; AAA, abdominal aortic aneurysm.
